# A split luciferase system for studying coronavirus M^pro^ dimerization *in vitro* and in living cells

**DOI:** 10.1016/j.jbc.2025.110890

**Published:** 2025-11-04

**Authors:** Renee Delgado, Jyoti Vishwakarma, Javier O. Sanlley Hernandez, Megan Tansiongco, Ashley Cuell, Agnieszka Dabrowska, Rahul Basu, Philipp A.M. Schmidpeter, You Hu, Susan E. Tsutakawa, Christina B. Cooley, Rommie E. Amaro, Reuben S. Harris

**Affiliations:** 1 Department of Biochemistry and Structural Biology, University of Texas at San Antonio, San Antonio, Texas, USA; 2 Department of Molecular Biology, University of California San Diego, La Jolla, California, USA; 3 Department of Chemistry, Trinity University, San Antonio, Texas, USA; 4 Howard Hughes Medical Institute, University of Texas at San Antonio, San Antonio, Texas, USA; 5 Department of Chemistry, University of Texas at San Antonio, San Antonio, Texas, USA; 6 Molecular Biophysics and Integrated Bioimaging, Lawrence Berkeley National Laboratory, Berkeley, California, USA

**Keywords:** coronavirus protease, protease, protease dimerization, protease inhibitor, SARS-CoV-2 main protease (M^pro^/3CL^pro^), split-luciferase biosensor system, virus replication

## Abstract

The main protease enzyme (M^pro^) of coronaviruses cleaves the viral polyprotein into functional units essential for virus replication. Prior work has demonstrated that M^pro^ functions as a homodimer. However, studies on the mechanism of dimerization have been challenging because the purified protease is mostly dimeric, dimerization-defective mutants lack proteolytic activity, and robust cell-based assays have yet to be reported. To enable work on M^pro^ dimerization, we have developed a quantitative luciferase-based SARS-CoV-2 (SARS2) M^pro^ biosensor that accurately reports protein dimerization in living cells and, upon purification, also *in vitro*. Co-transfection of cells with a construct expressing M^pro^ fused to the 18 kDa LargeBiT of luciferase (LgBiT) and a second construct with M^pro^ fused to the 1 kDa SmallBiT of luciferase (SmBiT) results in a reconstitution of luciferase activity in a dose-dependent manner that requires conserved residues within the dimerization interface. Proteolytic activity is dispensable for dimerization and, uniquely, a C145A catalytically inactive mutant exhibits enhanced dimerization signal likely due to lower cytotoxicity. M^pro^ enzymes from multiple different coronaviruses also dimerize in this system, indicating mechanistic conservation. Interestingly, this dimerization biosensor also provides a quantitative read-out of inhibitor-facilitated dimerization. Covalent SARS2 M^pro^ inhibitors such as nirmatrelvir cause a 3- to 5-fold increase in luciferase activity. Together with corroborating structural, biophysical, and molecular dynamics experiments, our studies support a model in which covalent M^pro^ inhibitors such as nirmatrelvir simultaneously block catalytic activity and induce allosteric stabilization of the dimeric complex.

Coronaviruses, including Severe Acute Respiratory Syndrome Coronavirus 2 (SARS-CoV-2; SARS2), are positive-sense RNA viruses with genomes of approximately 30 kb in size (1, 2). The viral genomic RNA encodes two overlapping polyproteins, pp1a and pp1ab, which are essential for viral transcription, replication, and pathogenesis. These polyproteins undergo proteolytic processing by two viral proteases to release individual functional proteins. The first, papain-like protease (PL^pro^), cleaves the viral polyproteins at 3 sites and, the second, main protease (M^pro^), cleaves the polyprotein at 11 conserved sites. Both proteases release their own polypeptides from the larger viral polyprotein as a likely first step in the overall proteolytic cascade required for virus replication (3).

As expected for essential viral enzymes, PL^pro^ and M^pro^ are both excellent drug targets. A variety of different PL^pro^ inhibitors are in various stages of development and have yet to reach the clinical setting [*e*.*g*., (4, 5)]. In contrast, due to fundamental work motivated by earlier coronavirus outbreaks (SARS-CoV and MERS), M^pro^ inhibitors have become validated therapeutics. For example, Paxlovid is used to minimize disease severity (6). Paxlovid combines nirmatrelvir, a reversible covalent M^pro^ inhibitor, with ritonavir, a CYP3A4 inhibitor, to protect the protease inhibitor from being metabolized rapidly. Similarly, Xocova was approved for emergency use during the COVID-19 pandemic in Japan (7). The active component of Xocova, ensitrelvir, is a non-covalent M^pro^ inhibitor with good stability *in vivo*. However, resistance mutations have been identified that may limit the use of both drugs. In the case of Xocova, a single amino acid substitution, M49I or M49L, severely compromises the ability of ensitrelvir to bind and inhibit M^pro^ (8, 9). Thus, there is a need for next-generation M^pro^ inhibitors that function independent of other drugs, are less susceptible to drug resistance mutations and, ideally, work independent of the protease’s active site.

The 33.8 kDa M^pro^ polypeptide dimerizes to yield a functional enzyme that cleaves the viral polyprotein into nonstructural proteins required for replication (10, 11). Two M^pro^ protomers assemble through interactions involving the N-terminal “N-finger” and domain III interface to form a catalytically competent enzyme where the dimerization interface forms an integral part of the substrate binding site, especially the S1 pocket. Dimerization, therefore, contributes to precisely positioning the catalytic dyad (Cys145 and His41) for efficient cleavage of the viral polyprotein.

Several approaches to studying M^pro^ dimerization exist, including analytical ultracentrifugation, mass-spectrometry, and small-angle X-ray scattering (SAXS) [*e.g*., (12, 13, 14)]. However, a robust human cell-based assay to study M^pro^ dimerization is lacking. Here, we describe a quantitative luciferase-based biosensor that accurately reports protein dimerization in living cells and, upon purification, also *in vitro.* Importantly, this biosensor also provides a quantitative read-out of inhibitor-facilitated dimerization *via* covalent small molecules such as nirmatrelvir. Recently, several reports on the stabilization of the dimerization interface have shown that covalent M^pro^ inhibitors, such as nirmatrelvir, GC376, and boceprevir, are able to shift the monomer-dimer equilibrium to primarily dimer (12, 15, 16). Using our luciferase-based M^pro^ dimerization biosensor, we can distinguish irreversible and reversible covalent inhibitors based on their mechanism of action at the catalytic cysteine, whereas non-covalent inhibitors do not facilitate dimerization. We propose a model in which covalent inhibitors mimic biological substrates by triggering the formation of a more stable dimer.

## Results

### A luciferase-based biosensor for quantification of M^pro^ dimerization in living cells

**Figure 1:**
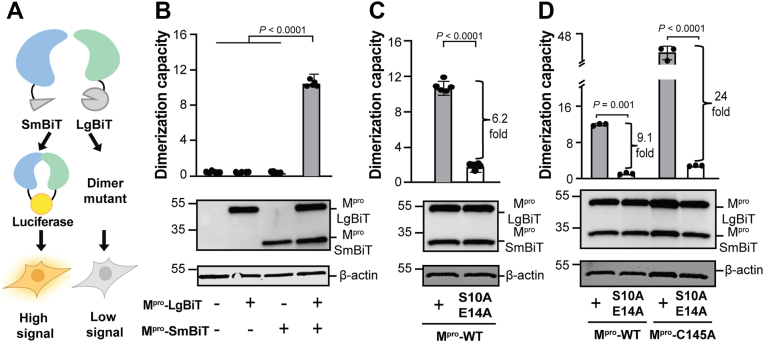
**Split-luciferase assay for SARS-CoV-2 M^pro^ dimerization.***A*, schematic of the split-luciferase biosensor for M^pro^ dimerization. M^pro^ is depicted fused to the small and large fragments of luciferase, SmBiT and LgBiT, respectively. Dimerization of M^pro^ enables luciferase reconstitution. *B–D*, Bar graphs showing dimerization capacity (luminescent signal x10^5^) of the indicated M^pro^ constructs expressed in 293T cells. Data are presented as mean ± SD from at least 2 independent biological replicates, each done in technical triplicate (*p*-values from unpaired two-tailed t-tests). Representative anti-M^pro^ immunoblots from a single experiment are shown below each graph with β-actin as a loading control.

The split luciferase system has been used to quantify interactions between a wide variety of different proteins in living cells [*e.g*., (17, 18)]. Knowing that the N-terminus of SARS2 M^pro^ forms part of the catalytic pocket and is not amendable to epitope tagging (19, 20), constructs were assembled in which the 18 kDa LargeBiT of luciferase (LgBiT) is fused to the C-terminus of one M^pro^-WT monomer and the 1 kDa SmallBiT of luciferase (SmBiT) is fused to the C-terminus of a second M^pro^-WT monomer (Fig. 1*A*). Transfection of either expression plasmid alone into 293T cells yields no luciferase signal (Fig. 1*B*). In comparison, co-transfection of M^pro^-LgBiT and M^pro^-SmBiT constructs yields a strong luciferase signal (Fig. 1*B*). In control experiments, immunoblots revealed that the M^pro^-SmBiT is not expressed as well as the M^pro^-LgBiT construct despite equimolar plasmid ratios during transfection. Co-transfection experiments with a range of molar ratios showed that a 1:1 protein expression ratio by immunoblot could be achieved by transfecting 16-fold more M^pro^-SmBiT plasmid (Fig. S1*A*). This 1:16 ratio of M^pro^-LgBiT to M^pro^-SmBiT is used in all experiments from here onward (Experimental Procedures). As expected from a transient transfection assay with optimized expression plasmids, the relative level of M^pro^ in this system is higher than that of native M^pro^ in infected cells but not grossly higher (Fig. S1*B*).

To ask whether the observed luciferase signal is due to M^pro^-dependent dimerization and concomitant luciferase reconstitution or simply due to luciferase reconstitution *in trans* (independent of M^pro^), a previously validated M^pro^-S10A-E14A dimerization-defective mutant was engineered into the M^pro^-LgBiT and M^pro^-SmBiT constructs and tested in parallel with wildtype constructs (21, 22). In comparison to the wild-type M^pro^ constructs, these two amino acid substitutions in M^pro^ resulted in 4- to 10-fold less luminescent signal (Fig. 1*C*). This important result indicates that the bulk of the observed luciferase signal is dependent on established dimer interface residues and that this system is a *bona fide* biosensor for M^pro^ dimerization.

Given positive results from co-expression in 293T cells, the M^pro^-WT-LgBiT and M^pro^-WT-SmBiT constructs were expressed in *E. coli*, purified to near homogeneity, and tested for dimerization activity *in vitro* (Experimental Procedures and Fig. S2, *A–C*). M^pro^-LgBiT and M^pro^-SmBiT exist in a dynamic equilibrium between monomeric and dimeric states. Upon mixing, the two subunits undergo a time-dependent association process that gradually reaches equilibrium, consistent with prior kinetic studies demonstrating that M^pro^ dimerization is not instantaneous but instead governed by a reversible, concentration-dependent equilibrium (12). This equilibration reflects the inherent structural plasticity of M^pro^ and underscores the transient nature of its dimerization interface. Upon mixing at a 1:1 M ratio, luciferase activity becomes detectable and continues to strengthen over time until an equilibrium is reached between the dissociation of the original proteins and the association into hetero-dimeric partners (Fig. S2*C*). Importantly, as above, most luminescent signal accumulation requires M^pro^ dimerization, as evidenced by S10A-E14A mutant proteins analyzed in parallel with the wild-type constructs (Fig. S2*C*).

### Inactive M^pro^-C145A catalytic mutant increases dimerization signal

We and others have shown that SARS2 M^pro^ overexpression is toxic to cells, likely by interfering with gene expression (23, 24, 25, 26). We therefore reasoned that disruption of the catalytic dyad through a C145A amino acid substitution might result in elevated luminescent signal in our cell-based dimerization system. In support of this idea, a single M^pro^-C145A amino acid substitution resulted in 2- to 3-fold higher luminescence signal compared to M^pro^-WT constructs (Fig. 1*D*). Therefore, to increase assay sensitivity and avoid confounding effects from cytotoxicity, structure-guided mutation studies of the M^pro^ dimerization mechanism leveraged M^pro^-C145A constructs in live cell studies. This approach is supported by SEC-MALS data showing that M^pro^-WT and M^pro^-C145A are similarly dimerization-proficient (Fig. S3).

### Mutational analysis of SARS2 M^pro^ dimer interface residues

**Figure 2:**
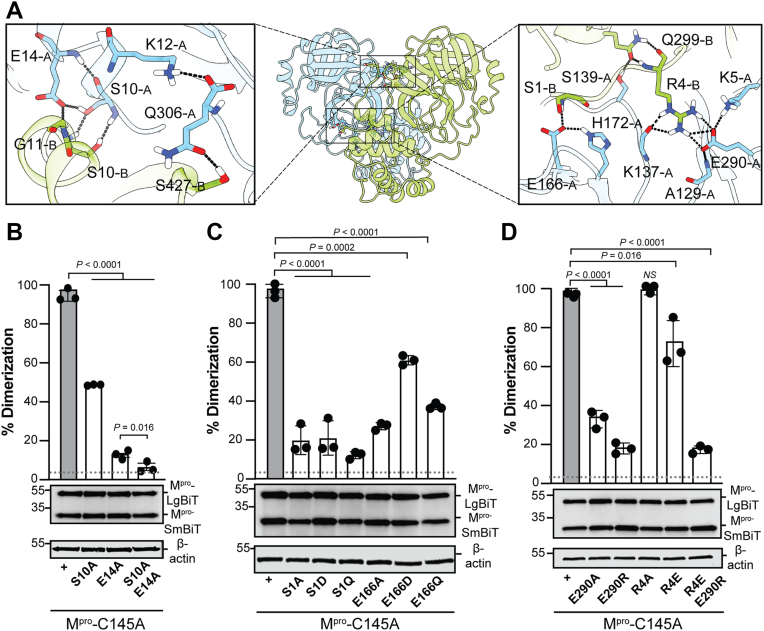
**Phenotypes of SARS-CoV-2 M^pro^ dimerization mutants.***A*, ribbon schematic of the SARS2 M^pro^ homodimer (PDB ID: 8BFQ). Residues subject to mutagenesis are labeled in the zoom-in panels to the *right* and *left* (top-down image of M^pro^). *Black dashes* indicate hydrogen bonds. *B–D*, Bar graphs showing dimerization capacity (luminescent signal x10^5^) of the indicated M^pro^-C145A derivatives expressed in 293T cells. The *gray dotted line* represents the dimerization level of the M^pro^-S10A-E14A double mutant. Data are presented as mean ± SD from at least 2 independent biological replicates, each done in technical triplicate (*p*-values from unpaired two-tailed t-tests). Representative anti-M^pro^ immunoblots from a single experiment are shown below each graph with β-actin as a loading control.

SARS2 M^pro^ is a dimer comprised of two protomers arranged nearly perpendicular to each other (10, 11, 27) (Fig. 2*A*). Each monomer contains three domains and features a catalytic dyad—His41 and Cys145—positioned between domains I and II (respectively, residues 10–99 and 100–182). The dimer interface is formed between domain III of monomer A and specific residues of monomer B, particularly within the “N-finger loop,” which play a crucial role in the dimerization process (13, 28, 29). Notably, residues S10 and E14, located in the α-helix of domain I, are conserved among various coronavirus proteases and contribute significantly to monomer–monomer interactions. Dimerization is essential for enzymatic activity, as the S1 residue of the N-finger of each monomer interacts with residue E166 of the opposite monomer, ensuring proper orientation of the S1 pocket within the substrate binding site. Interestingly, additional inter-subunit contacts also support dimer stability, such as a salt bridge between residues R4 and E290 (22, 30, 31).

To further validate the dimerization biosensor, we focused mutagenesis studies on these key inter-subunit contacts—S1, R4, S10, E14, E166, and E290. First, we examined the specific contributions of S10 and E14 to the interaction network within the N-finger loop of the protein (Fig. 2*A*). The results indicate that disrupting side chain interactions, particularly involving the conserved glutamate (E14A), leads to a pronounced loss of dimerization (Fig. 2*B*). In comparison, removal of the hydroxyl group of S10 (S10A) has a less dramatic impact on the backbone interactions at the dimer interface (Fig. 2*B*).

Second, we examined the contribution of residues that connect M^pro^’s dimerization interface and catalytic site. For instance, PDB structure 8BFQ indicates that the side chain of E166 (A protomer) forms H-bond interactions with the α-amine group with the backbone of S1 (B protomer; Fig. 2*A*). Consistent with the importance of this interaction, our results show that an alanine substitution at this position (E166A) reduces dimerization by 75%, shortening the residue by 1 carbon (E166D) drops dimerization by 30%, and changing the negatively charged glutamate to a non-charged polar residue of the same length (E166Q) affects dimerization by 60% (Fig. 2*C*). These results indicate that both side chain length and charge are necessary for maintaining the hydrogen bond network with serine 1, glutamate 166, and histidine 172. Similarly, changing serine 1 to any of the previously tested substitutions (S1A, S1D, or S1Q) results in a near-complete loss of dimerization, comparable to the S10A/E14A double mutant (represented by the grey dotted line in Fig. 2*C*). Given the similar phenotypes of these substitutions, it is likely that S1 has additional essential roles in M^pro^ function such as N-terminal processing and/or folding and maturation.

Finally, the dimerization biosensor was used to interrogate a salt-bridge interaction between R4 and E290; R4 is located near the N-terminus (N-finger) and E290 in the C-terminal region of the opposite monomer (Fig. 2*A*). These residues are involved in the inter-subunit electrostatic interactions that stabilize the dimer interface (21, 28, 32). Substituting the conserved residue glutamate 290 with either alanine or arginine (E290A, E290R) reduces dimerization to approximately 40% and 20% of M^pro^-C145A levels, respectively. Interestingly, an alanine (R4A) or a glutamate (R4E) substitution of arginine 4 had negligible or modest impact on dimerization, respectively (Fig. 2*D*). These results are consistent with the fact that E290 is conserved and R4 can vary between and within coronavirus species (see Fig. S4 for M^pro^ alignment). However, attempting to restore the disrupted salt bridge by combining the charge-reversing substitutions (R4E-E290R) did not rescue protein function. These results are consistent with X-ray structures showing that the negatively charged glutamate at position 290 makes multiple contacts and that both charge and side chain orientation are critical for function. In support of this, ITC experiments showed that recombinant M^pro^-R4A binds nirmatrelvir nearly as well as M^pro^-WT, whereas M^pro^-E290A showed no measurable binding (Fig. S5).

### Stabilization of M^pro^ dimeric state by covalent inhibitors

**Figure 3:**
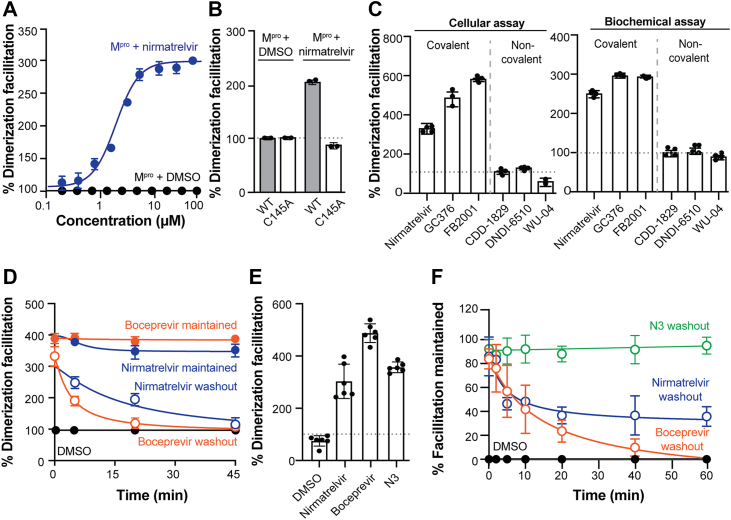
**Covalent inhibitors facilitate SARS2 M^pro^ dimerization.***A*, dose-dependent facilitation of M^pro^ dimerization in 293T cells by the indicated concentrations of nirmatrelvir. *B*, dimerization facilitation of M^pro^-WT *versus* M^pro^-C145A in 293T cells treated with DMSO or 15 μM nirmatrelvir. *Gray dashed line* denotes maximal signal without inhibitors (mean ± SD from at least 2 independent biological replicates, each done in technical triplicate). *C*, Bar graphs showing the dimerization capacity of M^pro^-WT against panel of covalent and noncovalent inhibitors in both cellular and biochemical assays (mean ± SD from at least 2 independent biological replicates, each done in technical triplicate). *D*, dimerization facilitation in 293T cells with or without inhibitor washout (mean ± SD from at least 2 independent biological replicates, each done in technical triplicate). *E*, Bar graphs showing dimerization facilitation by the indicated M^pro^ inhibitors in 293T cells (mean ± SD from at least 2 independent biological replicates, each done in technical triplicate). *F*, Dimerization facilitation with or without inhibitor washout in 293T cells (mean ± SD from at least 2 independent biological replicates, each done in technical triplicate).

The phenomenon of drug-facilitated dimerization has been described for coronavirus M^pro^ but it is not well understood mechanistically [*e.g*., (14, 15, 16)]. In general, it is thought that small molecule inhibitors such as nirmatrelvir, GC376, and boceprevir may shift the monomer:dimer equilibrium to favor the dimeric form. To ask whether the M^pro^ biosensor described above would be sensitive enough in living cells to report dimerization facilitation, a titration experiment was done with nirmatrelvir (Fig. 3*A*). Wild-type M^pro^-LgBiT and M^pro^-SmBiT constructs were co-transfected into 293T cells, replated 4 h post-transfection with varying concentrations of nirmatrelvir, and then incubated for an additional 44 h prior to quantification. In contrast to the DMSO-treated control, which continued to show steady dimerization signal, the luminescence of the nirmatrelvir-treated cells increased dose-responsively and reached approximately 300% of the control. Under these conditions, the half-maximal facilitation constant (FC_50_) for nirmatrelvir was 3.5 μM (Fig. 3*A*). Importantly, no dimerization facilitation was observed in experiments with C145A mutant constructs, demonstrating that the catalytic cysteine of M^pro^ is required (Fig. 3*B*).

A panel of validated SARS2 M^pro^ inhibitors was next surveyed for dimerization facilitation activity in living cells. Interestingly, consistent with the requirement for C145 described above, only covalent inhibitors – nirmatrelvir (33), GC376 (34), and FB2001 (35) were able to facilitate dimerization (Fig. 3*C*, left). In contrast, representative non-covalent inhibitors CDD-1829 (36), DNDI-6510 (37), and WU-04 (38), were unable to facilitate dimerization (Fig. 3*C*, left). Similar results were found with recombinant protein dimerization *in vitro* (Fig. 3*C*, right). Together, these results combine to indicate that the property of dimerization-facilitation is likely intrinsic to the nature of the inhibitor and to M^pro^ itself.

Next, washout experiments were performed to address whether the dimerization phenotype is reversible. As anticipated, well-studied covalent inhibitors nirmatrelvir and boceprevir were able to maintain maximal dimerization facilitation if each inhibitor was maintained in the growth medium (Fig. 3*D*). However, upon washout and replacement with inhibitor-free growth medium (with DMSO as a control), the facilitation effect rapidly dissipated with near baseline dimerization levels re-established within 30 to 45 min (Fig. 3*D*). These results are consistent with the mechanisms of M^pro^ inhibition by nirmatrelvir and boceprevir, as both are reversible covalent inhibitors (39, 40). To further interrogate the mechanism of dimerization facilitation, we also tested an irreversible covalent inhibitor N3 (19). The inhibitor N3 is a peptidomimetic Michael acceptor that mimics the natural substrate of SARS2 M^pro^. Upon binding in the active site, its peptide backbone fits into the substrate pocket, positioning an electrophilic acrylate for nucleophilic attack by the catalytic cysteine (Cys145). We found that N3 is a strong dimerization facilitator and that the facilitation phenotype is not reversed by inhibitor-free growth medium (Fig. 3, *E* and *F*). These experiments indicate that the M^pro^ dimerization biosensor can be used to study the unique mechanism of dimerization facilitation and that the washout assay may also be useful for inhibitor classification as non-covalent, reversible covalent, or irreversible covalent inhibitors.

### Conservation and mechanism of dimerization facilitation

**Figure 4:**
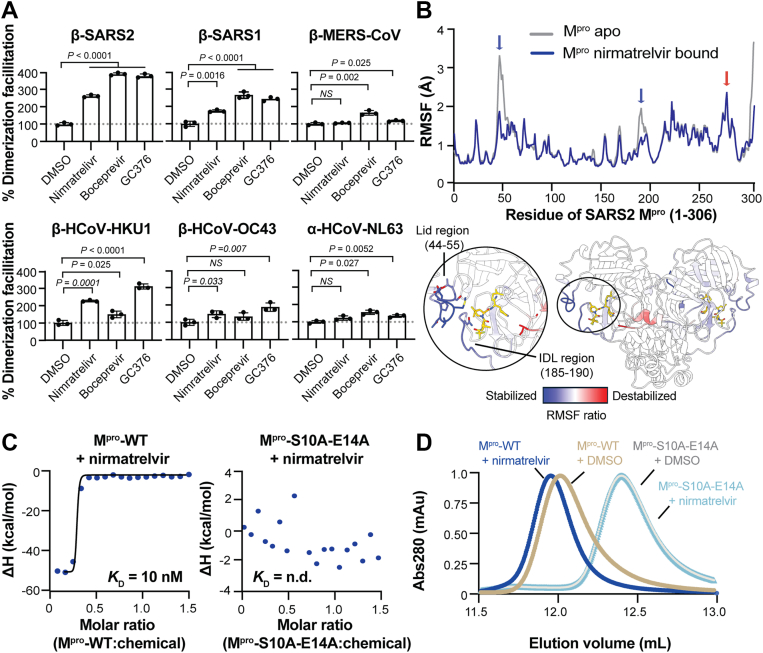
**Mechanistic conservation and nirmatrelvir-mediated M^pro^ dimer stabilization.***A*, Bar graphs showing dimerization facilitation by the indicated inhibitors for the M^pro^ enzymes of the indicated coronaviruses (mean ± S.D. from at least 2 independent biological replicates, each measured in technical triplicate). *B*, RMSF of SARS2 M^pro^ residues (1–306) in apo *versus* nirmatrelvir-bound states. Regions of stabilization and destabilization are highlighted with *blue* and *red* arrows, respectively (*upper panel*) and mapped onto the M^pro^ structure lid and IDL regions highlighted (*lower panel*). *C*, ITC data for nirmatrelvir binding to M^pro^-WT (*left*) and M^pro^-S10A-E14A (*right*; n.d., not determinable). *D*, SEC profiles of M^pro^-WT and M^pro^-S10A-E14A in the presence of DMSO (control) or nirmatrelvir.

To gain deeper insights into dimerization facilitation, mechanistic conservation was assessed by testing a panel of M^pro^-SmBiT and M^pro^-LgBiT constructs representing five different β-coronaviruses and one α-coronavirus (Fig. 4*A*). For these studies, we used the reversible covalent inhibitors nirmatrelvir, boceprevir, and GC376 as mechanistic probes, because the latter two compounds are known to be promiscuous (34, 41). All tested M^pro^ enzymes from β-coronaviruses, including SARS2, SARS-CoV-1, MERS-CoV, HCoV-HKU1, and HCoV-OC43, as well M^pro^ from the α-coronavirus HCoV-NL63, exhibited dimerization facilitation to varying extents (Fig. 4*A*). Moreover, in the few instances where facilitation was not observed, such as nirmatrelvir against MERS-CoV or HCoV-NL63 and boceprevir against HCoV-OC43, prior studies have already shown that these compounds are ineffective against these M^pro^ enzymes (42, 43). In other words, dimerization facilitation, like dimerization itself, appears to be a fundamental and conserved property of coronavirus M^pro^ enzymes.

To further probe the mechanism of inhibitor-facilitated dimerization, we compared all-atom MD simulations of the SARS2 M^pro^ dimer in apo and nirmatrelvir-bound states. Per-residue root mean square fluctuation (RMSF) analyses of the resulting MD trajectories revealed two prominent regions with greater mobility (destabilization) in the absence of nirmatrelvir – the lid and the inter-domain loop (IDL) regions (Fig. 4*B*). The lid region spans residues 44 to 55, and it directly contributes multiple interactions to the substrate/inhibitor binding pocket. Indeed, structural studies have shown that changes in the lid region often occur coincident with inhibitor binding (19, 23). The IDL region forms part of the opposite side of the active site and contributes to inhibitor binding (12, 19, 27). These observations indicate that the nirmatrelvir-bound state is more stabilized (compact) than the apo-form of the protein. Consistent with this model, nirmatrelvir readily binds to M^pro^-WT but not to the monomeric M^pro^-S10A-E14A double mutant as evaluated by ITC and SEC-MALS experiments (Fig. 4, *C* and *D*). For instance, treatment with nirmatrelvir shifts the SEC profile of M^pro^-WT (but not M^pro^-S10A-E14A), likely reflecting a more compact and stabilized conformation upon binding of the drug (Fig. 4*D*).

### Covalent inhibitor, nirmatrelvir, stabilizes the M^pro^ dimer

**Figure 5:**
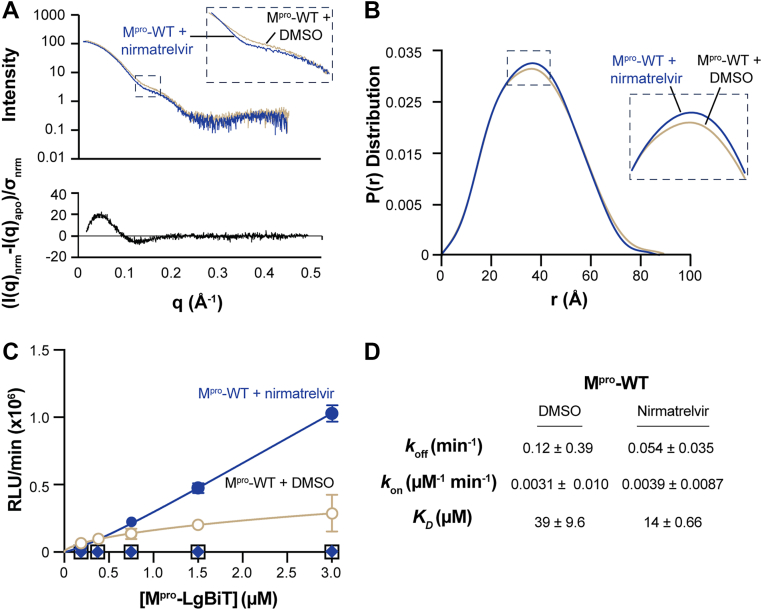
**Nirmatrelvir stabilizes the SARS2 M^pro^ dimer.***A* and *B*, SEC-SAXS analysis of M^pro^-WT. Reciprocal space scattering profiles and residuals in *panel A* indicate a more globular shape in the presence of nirmatrelvir, with inset highlighting a change in one of the scattering profile regions. Pair distance distribution function [P(r)] in *panel B* derived from SAXS data shows a slightly more compact structure in the presence of nirmatrelvir, with inset showing a change in peak position. *C*, association kinetics of M^pro^-WT-SmBiT or M^pro^-S10A-E14A-SmBiT with increasing concentrations of the respective M^pro^-LgBiT constructs in the presence or absence of nirmatrelvir. M^pro^-S10A-E14A association kinetics did not increase above baseline levels in the presence or absence of nirmatrelvir *(blue diamonds* and *open squares*). *D*, *k*_off_, *k*_on_, and *K*_D_ for M^pro^ in the presence or absence of nirmatrelvir (mean ± SD; n = 2 independent experiments). Kinetic parameters are determined by fitting binding data in *panel C* with the Hill slope equation. Kinetic data for M^pro^-S10A-E14A could not be determined.

To directly interrogate the solution conformation of M^pro^ and assess the impact of nirmatrelvir, SAXS experiments were performed with the protein incubated with either nirmatrelvir (in DMSO) or a DMSO-only control (Figs. 5 and S6). The protein eluted as a single major peak consistent with a dimeric form. While no distinct monomer peak was observed, a shoulder in the DMSO sample might indicate a small monomeric population. Multi-angle light scattering and SAXS curve calculations confirmed both samples were dimeric. Overall, the curves were similar, but subtle differences were visible in the residual plot and the zoomed-in insets of both reciprocal and real space SAXS curves (Fig. 5*A*). Specifically, the DMSO control-treated protein appeared more extended than the nirmatrelvir-bound protein (Fig. 5*B*). We investigated the consistency of atomic models, built from M^pro^ monomers or dimers observed in the crystallographic lattice, with individual scattering curves. The nirmatrelvir-incubated protein showed good correspondence with nirmatrelvir-bound crystal dimers. Interestingly, the DMSO data were best explained by introducing a significant population of an improbable dimer, based on PDB:7UJ9 (Fig. S6). This suggests that the WT protease, in the absence of nirmatrelvir, adopts an open conformation for which the atomic structure is unknown. While no monomer population was observed, this supports the hypothesis that the WT protein forms an unstable, open dimer that is subsequently converted to a stable, globular dimer upon nirmatrelvir binding.

Finally, the biochemical SARS2 M^pro^ dimerization assay was applied to quantify association and dissociation parameters. In this system, luminescence results from the dimerization of M^pro^-LgBiT and M^pro^-SmBiT and increases as a function of protease concentration. Binding curves were fit using the ‘specific binding with Hill slope’ equation, which relates the luminescent signal to the fraction of dimer formed. From these fits, apparent dissociation constants *K*_D_ were obtained, providing a quantitative measure of M^pro^ dimerization in the absence or presence of nirmatrelvir (Fig. 5*C*). For M^pro^-WT dimerization, this analysis yielded a *K*_D_ of 39 ± 9.6 μM, consistent with reported values [*e.g*., (13, 44)] (Fig. 5*D*). Addition of nirmatrelvir to M^pro^-WT resulted in a steeper Hill slope and a nearly 3-fold lower dimerization *K*_D_, 14 ± 0.66 μM, indicating enhanced dimerization and a slower off-rate. The shift toward tighter binding, combined with kinetic analyses revealed a decreased apparent off-rate, indicating that nirmatrelvir stabilizes the M^pro^ dimer in a binding-competent conformation that simultaneously decreases the likelihood of protomer dissociation. For the M^pro^-S10A-E14A mutants, we were unable to determine a reliable dissociation constant (*K*_D_) because these protomers fail to dimerize.

## Discussion

Coronavirus M^pro^ functions as an obligate homodimer. Here, using SARS2 M^pro^ as a primary example, we report a coronavirus main protease dimerization assay that enables quantitative interrogation of mutants and provides a tractable system to investigate mechanisms of dimerization facilitation both in cells and, upon purification, *in vitro*. Importantly, this approach addresses a critical gap in the coronavirus protease field, where robust methods for monitoring M^pro^ dimerization rapidly in living cells have previously been limited. By creating a robust, scalable, and broadly applicable M^pro^ dimerization system, our studies expand the experimental toolkit available for dissecting M^pro^ regulation and may accelerate efforts to fully define how dimerization governs protease activity.

A major strength of our M^pro^ dimerization biosensor is the fact that mutants can be constructed and assessed rapidly through simple co-transfection experiments. For instance, we find that conserved M^pro^ residues such as S1, E14, and E290 are critical for dimerization, consistent with prior reports using biophysical assays [*e.g*., (21, 23, 29, 45)]. Other residues such S10, E166, and R4 are less critical. Importantly, these mutational studies not only validate this assay system, but also suggest that it may be used in future studies to screen for additional formative mutants as well as chemical inhibitors of dimerization.

Another key strength of the M^pro^ dimerization system reported here is that it can also be used to study covalent inhibitor-facilitated dimerization. For example, established covalent inhibitors such as nirmatrelvir, boceprevir, and GC376 readily facilitate SARS2 M^pro^ dimerization. Moreover, adding a washout step to the workflow confirms that these are all reversible covalent inhibitors. In contrast, at least one reported covalent inhibitor, N3, facilitates dimerization in an irreversible manner (19). Nirmatrelvir’s nitrile engages the catalytic cysteine (Cys145) to form a covalent bond through a thioimidate linkage, which can reverse to regenerate the free thiol of Cys145 and the intact inhibitor. By contrast, the Michael acceptor of N3 undergoes nucleophilic attack by Cys145, forming an irreversible covalent thioether bond to M^pro^. Thus, the M^pro^ dimerization biosensor described here can be used to rapidly distinguish between non-covalent, reversible covalent, and irreversible covalent inhibitors.

Taken together, our results suggest a model in which covalent inhibitors such as nirmatrelvir facilitate dimerization by triggering a network of allosteric changes that result in a hyper-stabilized dimeric conformation. Facilitation minimally requires covalent bond formation between the inhibitor and C145. Moreover, dimerization facilitation is apparent using a panel of diverse β- and α-coronavirus M^pro^ enzymes, indicating a conserved mechanism akin to dimerization itself. We have yet to identify a covalent inhibitor that fails to facilitate dimerization, or a mutant that loses susceptibility to inhibitor-mediated facilitation. Additional studies will be needed to further address the mechanism of dimerization facilitation.

## Experimental procedures

### Plasmids

Omicron variants of SARS2 M^pro^ including BA5 (GenBank: OP054053) have a histidine at position 132, whereas original alpha variants such as WA1 (GenBank: MT246667) have a proline. Gene blocks were designed to incorporate the Omicron M^pro^ sequence into the pcDNA5/TO expression plasmid using HindIII/Not1 digestion. Each gene block has a HindIII site, a Kozak sequence (GCCACC), an Omicron M^pro^ coding sequence (GenBank: OP054053), a flexible linker (5′-ggcccgggcgggggaggttcgggaggtagctca), the small (SmBiT) or large (LgBiT) split-luciferase fragment, a stop codon, and a Not1 site. Gene blocks were hydrated in molecular-grade water (100 ng/μl), ligated into the pJET1.2/blunt cloning vector, and transformed into *E. coli* DH10α. Positive clones were miniprepped and verified by Sanger sequencing. The HindIII/NotI fragments were then subcloned into similarly digested pcDNA5/TO vector and reconfirmed by DNA by restriction analysis and Sanger sequencing. Derivative plasmids were created by site-directed mutagenesis (primer sequences in Table S1). All other tested M^pro^ constructs of β-coronaviruses including SARS-CoV-1 (GenBank: AAP41036), MERS-CoV (GenBank: AFV09327), HCoV-HKU1 (GenBank: YP459936), and HCoV-OC43 (GenBank: YP_009555238), and the α-coronavirus HCoV-NL63 (GenBank: YP_003766.2) were ordered as gene blocks from IDT with HindIII and NotI sites for restriction digestion and ligation into similarly cut pcDNA5/TO.

### Cell culture for dimerization assay

293T cells (ATCC CRL-3216) were cultured in DMEM, high glucose, pyruvate (Thermo Fisher Scientific, 11-995-073), supplemented with 10% fetal bovine serum (FBS; Biowest, 058N24) and 1% penicillin-streptomycin (P/S; Thermo Fisher Scientific, 15140163) at 37 °C in a humidified incubator with 5% CO_2_. Cells were passaged at 70 to 80% confluence with a single PBS wash (Thermo Fisher Scientific, BP39920), treatment with 0.05% Trypsin-EDTA (1X, phenol red; Genesee Scientific 25-510F) until detachment, and resuspension in complete medium.

#### For dimerization studies:

3 × 10^5^ 293T cells were seeded in a 6-well plate and transfected 24 h later with 50 ng and 800 ng of WT or C145A M^pro^-LgBiT and M^pro^-SmBiT constructs, respectively, using the manufacture’s protocol (TransIT-LT1, Mirus Bio). 48 h post-transfection, cells were washed once with PBS, trypsinized, resuspended, and counted. Cells were diluted to yield a suspension containing 5 × 10^5^ cells/ml, and 100 μl of the suspension was plated into each well of a white flat 96-well plate yielding a final of 5 × 10^4^ cells/well. Then, 25 μl of a 20 × dilution of Nano-Glo reagent (Promega, N1110) was added directly on top of the 100 μl cell suspension, followed by a 5 min incubation before measuring luminescence on a Tecan Spark multimode plate reader. Percent dimerization was calculated for each mutant using the following equation (RLU, raw luminescent units):

$$\%\;Dimerization=100\;x\frac{RLU\;of\;mutant}{RLU\;of\;WT}$$

Leftover cell lysates were harvested for immunoblots (below).

#### For dimerization facilitation studies:

3 × 10^6^ 293T cells were seeded in a 10 cm dish and transfected 24 h later with 300 ng and 4800 ng of WT M^pro^-LgBiT and M^pro^-SmBiT constructs (1:16), respectively. 4 h post-transfection, cells were washed once with PBS, trypsinized, resuspended, and counted. Cells were diluted to yield a suspension containing 1.0 × 10^6^ cells/ml, and 50 μl of the suspension was plated into a 96-well plate with 50 μl of media containing 2 × the desired inhibitor concentration yielding a final 1 × inhibitor concentration and 5 × 10^5^ cells/well. 48 h after plating into 96-well plates, 25 μl of a 20× dilution of Nano-Glo reagent was added directly on top of the 100 μl cell suspension, followed by a 5 min incubation before measuring luminescence on a Tecan Spark multimode plate reader. Percent facilitation was calculated for each inhibitor using the following equation (RLU, raw luminescent units):

$$\%\;Facilitation=100\;x\frac{RLU\;of\;WT\;Mpro+inhibitor}{RLU\;of\;WT\;Mpro+DMSO}$$

Leftover lysates were used for immunoblots (below).

### Leftover lysates were used for immunoblots (below).

All M^pro^ inhibitors tested here target the active site pocket based on crystallographic, biophysical, biochemical, and genetic studies. M^pro^ inhibitors were purchased as powders and dissolved in DMSO at working concentrations of 10 mM. Nirmatrelvir (33), boceprevir (46), GC376 (34), WU-04 (38), and N3 (19) were obtained from MedChemExpress (catalog no. HY138687, HY10237, HY100721, HY149535, and HY136149, respectively). CDD-1829 and FB2001 were reported (36, 47) and provided as fresh powders by Rayhan Biswas and Daniel Harki (University of Minnesota). DNDI-6510 (37) was provided by Annette von Delft (Oxford University). The integrity of all of these M^pro^ inhibitors was verified using an established biochemical assay with untagged SARS2 Omicron M^pro^ and a model fluorescent peptide substrate (48), and all IC_50_ values were 2-fold of published values (see references above).

### Immunoblots

Transfected 293T cells were pelleted by centrifugation and lysed in RIPA buffer (10 mM Tris-HCl, pH 8.0; 1 mM EDTA; 0.5 mM EGTA; 1% Triton X-100; 0.1% sodium deoxycholate; 0.1% SDS; 140 mM NaCl) supplemented with 1× cOmplete Protease Inhibitor Cocktail, EDTA-Free (Sigma-Aldrich, 11836170001). Total protein concentrations were determined using a BCA Protein Assay Kit (Thermo Fisher Scientific, 23227) according to the manufacturer’s instructions. Samples were diluted as needed to achieve a final loading amount of 20 μg per well. Protein samples were mixed with 2× SDS-PAGE loading buffer (0.1 M Tris-HCl, pH 6.8, 20% glycerol, 4% SDS, 200 mM dithiothreitol [DTT], and 0.01% Orange G) and denatured at 95 °C for 5 min, fractionated using SDS-PAGE (4–20% Mini-Protean gel; Bio-Rad, 4568093), and transferred to a polyvinylidene difluoride (PVDF) membrane (Millipore Sigma, IPVH00010). Immunoblots were probed with rabbit anti-SARS-CoV-2 3C-like protease (1:1000; Cell Signaling Technology, 51661) and mouse anti-β-actin (1:10,000; Cell Signaling Technology, 4967) followed by anti-mouse IgG IRDye 680 (1:10,000; LICORbio, 926-68070) and anti-rabbit IgG-horseradish peroxidase (HRP; 1:10,000; Jackson ImmunoResearch Laboratories, 111-035-144). HRP secondary antibody was visualized using the SuperSignal West Femto maximum sensitivity substrate (Thermo Fisher Scientific, PI34095). Images were acquired using the LICORbio Odyssey Fc imaging system.

### Sequence alignments

Coronavirus main protease amino-acid sequences were obtained from Protein Data Bank entries 8HOL (SARS2), 1Q2W (SARS-CoV), 5C3N (MERS-CoV), 9C7W (HCoV-OC43), 7E6R (HCoV-NL63), 3D23 (HCoV-HKU1). Sequences were aligned using the NCBI BLASTp multiple alignment tool with default parameters. Alignments were exported in Clustal format for analysis and figure preparation.

## *M*^*pro*^*purification*

Gene blocks were designed for Golden Gate assembly (New England Biolabs, R0739S) into the pE-SUMO expression plasmid (49) using BsaI digestion. Each gene block has a BsaI site, Omicron M^pro^ coding sequence (GenBank: OP054053), a flexible linker (5′-ggcccgggcgggggaggttcgggaggtagctca), the small (SmBiT) or large (LgBiT) split-luciferase fragment, a stop codon, and another BsaI site. Gene blocks were hydrated in molecular-grade water (100 ng/μl), ligated into the pJET1.2/blunt cloning vector, and transformed into *E. coli* DH10α. Positive clones were miniprepped and verified by Sanger sequencing. The BsaI fragments were then subcloned into similarly digested pE-SUMO vector and reconfirmed by DNA by restriction analysis and Sanger sequencing. All M^pro^ mutants were generated as above by site-directed mutagenesis (primers in Table S1) and confirmation by Sanger sequencing.

*N*-terminal His_6_-Sumo-M^pro^-WT and mutant constructs were expressed in *E. coli* BL21(DE3) (New England Biolabs, C2527H). A single colony was grown overnight to saturation in 25 ml LB medium supplemented with 100 mg/ml carbenicillin (Thermo Fisher Scientific, J6194903). 1% of this primary culture was used to inoculate 1 L of LB supplemented with 100 mg/ml of carbenicillin and incubated at 37 °C and 180 rpm. Once the optical density (OD) reached 0.6, the culture was induced with 0.5 mM IPTG (Thermo Fisher Scientific, 15529019) and the temperature lowered to 18 °C for an additional 20 h. The bacteria were collected by centrifugation at 6000*g*, resuspended in 50 mM Tris, pH 8.0, 250 mM NaCl, 5 mM β-mercaptoethanol (Thermo Fisher Scientific, O33461–100), 5 mM imidazole (Thermo Fisher Scientific, A1022122), and 5% glycerol (Thermo Fisher Scientific, A16205AP), and lysed by sonication. M^pro^ was captured from cleared lysate using a nickel-nitrilotriacetic acid gravity flow affinity column (Thermo Fisher Scientific, R90115), washed by a gradient of imidazole, and eluted with 300 mM imidazole. The eluted fractions of M^pro^ were pooled together and treated with Ulp1 Sumo-protease to remove the 6XHis-Sumo tag and further purified by gel filtration using HiLoad 26/600 Superdex 200 prep-grade (pg) column (Cytvia, 28989336) in 20 mM Tris-HCl, pH 8.0, 150 mM NaCl, 0.5 mM TCEP. The peak fractions showing M^pro^ in SDS-PAGE were pooled and concentrated to 4 mg/ml as determined by UV absorbance (NanoDrop 8000 spectrophotometer), and flash frozen in liquid nitrogen for long-term storage at −196 °C and usage in biophysical, structural, and biochemical experiments.

### Size-exclusion chromatography multi-angle light scattering (SEC-MALS)

All recombinant M^pro^ protein preparations were examined by SEC-MALS to determine molar masses in solution. SEC-MALS was performed on a Wyatt DAWN 8 device connected to an Agilent Affinity FPLC using a Waters XBridge Premier Protein SEC Column (250 Å, 7.8 × 300 mm, 2.5 μm particle size; Cat. No. 186009962) and a refractometer-Optilab (Wayatt). The column was pre-equilibrated in SEC buffer (20 mM Tris-HCl pH 7.5, 100 mM NaCl, 0.5 mM TCEP and 2.5% DMSO) with a flow rate of 0.5 ml/min, and 20 μl of protein sample (4 mg/ml for each protein) was loaded onto the column. For nirmatrelvir-bound samples, 600 uM nirmatrelvir was added to M^pro^-WT and M^pro^-S10A/E14A, mixed and incubated on ice for 30 min. Samples were centrifuged at 12,000 rpm for 15 min at 4 °C, before injection. UV absorbance was monitored at 280 nm. Data processing and molar mass calculations were performed using the ASTRA software (Wyatt Technology).

### In vitro dimerization experiments

#### For dimerization studies

Dimerization reactions were carried out in 50 μl reactions in Greiner 96-well chimney half-area plates (Greiner Bio-One, 675076) with 50 nM of each M^pro^-SmBiT and M^pro^-LgBiT protein, 20 mM Tris-HCl, pH 8.0, 150 mM NaCl, 1 mM EDTA, 0.05% Tween20, 0.1 mg/ml bovine serum albumin (BSA), 1 mM DTT. Then 12.5 μl of a 20× dilution of Nano-Glo reagent (Promega, N1110) was added directly on top of the 50 μl suspension. Luminescence was measured once per minute using a Tecan Spark 10 M plate reader until equilibrium was reached.

#### For dimerization facilitation studies

Dimerization facilitation reactions were carried out in 50 μl reactions in Greiner 96-well chimney half-area plates (Greiner Bio-One, 675,076) with 50 nM of each M^pro^-SmBiT and M^pro^-LgBiT protein, 20 mM Tris-HCl, pH 8.0, 150 mM NaCl, 1 mM EDTA, 0.05% Tween20, 0.1 mg/ml bovine serum albumin (BSA), 1 mM DTT. Either M^pro^-LgBiT or M^pro^-SmBiT split protein was incubated at room temperature with none, or various concentrations of chemical (2-fold serial dilution series starting at 100 μM) for 30 min in reaction buffer prior to addition of 25 μl of either M^pro^-LgBiT or M^pro^-SmBiT. 12.5 μl of a 20× dilution of Nano-Glo reagent (Promega, N1110) was added directly on top of the 50 μl suspension. Luminescence was measured once per minute using a Tecan Spark 10 M plate reader until equilibrium was reached.

### Isothermal titration calorimetry (ITC) experiments

Binding experiments were performed on a MicroCal PEAQ-ITC (Malvern Panalytical) at 25 °C. The M^pro^-WT, S10A/E144A, R4A, and E290A proteins without any tag were diluted in ITC buffer (20 mM Tris-HCl, 8.0, 100 mM NaCl, 0.5 mM TCEP, and 1% DMSO) to a final concentration of 10 μM. 300 μl of 10 μM M^pro^ was placed in a calorimetric cell, and the syringe was loaded with 70 μl of 100 μM of nirmatrelvir. The reference cell was filled with 300 μl MilliQ water. 25 injections of 1.5 μl compounds were injected into the cell at intervals of 150 s with a stirring speed of 750 rpm. To measure the binding affinity (*K*_D_), Enthalpy (ΔH), Entropy (ΔS), and stoichiometry (n), the raw ITC data were analyzed using MicroCal PEAQ-ITC analysis software.

### M^pro^ dimerization kinetics

#### K_D_ determination

First, M^pro^-LgBiT was titrated starting at 3 μM, 12-point, 2-fold dilution across in the reaction plate in 25 μl total volume. Second, 25 μl of M^pro^-SmBiT was added, for a final concentration of 20 nM. Third, 12.5 μl of a 20× dilution of Nano-Glo reagent (Promega, N1110) was added directly on top of the 50 μl suspension. Last, luminescence was measured once per minute using a Tecan Spark 10 M plate reader until equilibrium was reached. Dose–response curves (signal vs. total [M^pro^-LgBiT]) were fit in GraphPad Prism 10.1.0 using Specific binding with Hill slope where B_max_ = Plateau signal, K_D_ = apparent dissociation constant, and *h* = hill slope:

$$Y=\frac{B_{\max}*X^{h}}{K_{D}^{h}+X^{h}}$$

#### k_on_/k_off_ determination

Proteins were diluted serially in separate 96-well plates, with M^pro^-LgBiT diluted two-fold across 12 columns beginning at 3 μM and M^pro^-SmBiT diluted two-fold down 8 rows beginning at 500 nM. Equal volumes (25 μl) from the corresponding wells of each dilution series were then combined in a new 96-well plate to generate a two-dimensional concentration matrix for kinetic analysis. Reactions were initiated by addition of 12.5 μl of a 20 × dilution of Nano-Glo reagent directly to each well, and luminescence (RLU) was continuously recorded as a function of time. The resulting time courses for [M^pro^-SmBiT] + [M^pro^-LgBiT] were fit using GraphPad Prism 10.1.0 “Association kinetics – Two or more concentrations of hot ligand” model to determine association rate constants (*k*_on_; μM^−1^ min^−1^) and dissociation rate constants (*k*_off_; min^−1^):

$$K_{D}=\frac{k_{off}}{k_{on}}$$

### Molecular dynamics (MD) simulations

MD simulations sample the statistical distributions of protein ensembles by integrating Newton’s equations of motions over femtosecond timesteps, generating trajectories that allow the modeling of conformational changes of proteins at biologically relevant timescales. To better understand the structural role of dimerization facilitation, microsecond scale all-atom molecular dynamics (MD) simulations of M^pro^ homodimer in the apo and nirmatrelvir-bound state were done. The crystal structure of SARS2 M^pro^ in complex with nirmatrelvir (PDB: 7VH8) was used for building the apo and ligand bound models (50). Apo M^pro^ was constructed by removing the ligand from the covalently bound C145. The hydrogen atoms in the structures were modeled by running the Protein Preparation protocol using the Maestro program developed by Schrödinger (51).

Additionally, the protonation states of titratable residues were calculated for a target pH of 7.4 using the PROPKA server (52, 53). The parametrization (*antechamber)*, solvation (*tLEaP*), and MD simulations (*pmemd.cuda)* of protein-ligand complexes in explicit water solvent at physiological conditions (300 K, 1 atm, 0.150 M NaCl, pH = 7.4) were performed using the suite of programs developed by AmberTools23. The antechamber package was used for the parametrization of the covalently bound C145 residues using the am1-bcc charge method (54). The ff19SB force fields was used to describe all protein atoms in conjunction with the OPC water model (54, 55). Solvation consisted of a truncated octahedron water box built around the protein with a 10 Å distance cutoff using *tLEaP*. Sufficient Na^+^ and Cl^-^ ions were randomly placed in the water box to match a final concentration of 0.15 M. Additionally, sufficient counterions were added to neutralize protein charge.

System minimization, heating, and equilibration stages were performed to adequately prepare the model before production dynamics. Harmonic restraints were applied where necessary to selectively restrain atomic motions of selected atoms during the aforementioned stages. Additionally, periodic boundary conditions were used with a 2 fs timestep in between frames. The SHAKE algorithm was used to constrain all bonds between hydrogens and heavy atoms to their equilibrium distance. Minimization was divided into five stages where restraints on non-hydrogen atoms, protein atoms, backbone protein atoms, and ligand were gradually removed before conducting an unrestrained 2000 cycles of steepest descent, respectively. Unrestrained minimization consisted of 36,000 steps of conjugate gradient minimization algorithms. Heating to target physiological temperature (310 K), consisted of 250 ps of heating with restraints followed by 250 ps of unrestrained heating. Following heating, the system was then allowed to equilibrate by running a 1 ns at a constant pressure of 1 atm. Equilibration was confirmed by plotting backbone root mean square deviation (RMSD) over the course of equilibration against the starting structure. From the equilibrated structure, production runs consisted of 1 μs constant pressure simulations in triplicates. Trajectories were analyzed using a combination of CPPTRAJ and MDAnalaysis packages. To assess the stability of the system over the course of the simulation, we calculated the Root Mean Square Deviation (RMSD) over the course of the trajectory as an average of all three replicates. Additionally, the Root Mean Square Fluctuation (RMSF) was calculated for every residue in the protein. Comparison of RMSF values allows us to evaluate residues that are stabilized or destabilized by ligand binding.

### SEC-MALS small-angle X-ray scattering (SEC-MALS-SAXS)

SEC-MALS-SAXS data were collected on SARS2 M^pro^-WT on the SIBYLS beamline 12.3.1 at Advanced Light Source (ALS) at Lawrence Berkeley National Laboratory (Table S2). The X-ray wavelength was 1.24 Å and a sample-to-detector distance of 2100 mm. 60 μl of each M^pro^ (12 mg/ml) samples was injected to a Shodex 802.5 column equilibrated with running buffer (20 mM Tris-HCl pH 7.5, 200 mM NaCl, 1 mM DTT). The column was run at a flow rate of 0.65 ml/m, and 2 s X-ray exposures were recorded continuously for a 25 m elution. BioXTAS RAW (56) was used for SEC-SAXS data buffer subtractions and data reduction. Based on the MALS chromatogram, the SAXS frames recorded before or after the protein elution peak were used to subtract all other frames. Guinier plots were linear, indicating no detectable non-specific aggregation. The maximal inter-particle dimension was calculated by pair distribution function [P(r)] using GNOM. We built atomic models of M^pro^ dimers using symmetry mates of a crystal structure of M^pro^ (PDB: 6Y2E) with PyMOL (Schrödinger) and modelling missing loops of published M^pro^ structures (PDB: 8HOL, 8BFQ, 8DZ2, 7TLL) through modeler comparative in UCSF ChimeraX (57, 58). One of the generated 6Y2E dimer was used to explore structural flexibility by AllosMod (59). In MultiFoXS, SAXS profiles for the thirteen generated models were calculated and compared to the experimental SAXS data individually or as ensembles (60, 61). Structures were drawn with PyMOL and ChimeraX.

## Data availability

Data generated in this study are contained within the article. Raw data are available by email request to rsh@uthscsa.edu.

## Supporting information

This article contains supporting information (Supplementary Tables S1 and S2 and Figures S1–S6).

## Conflict of interest

The authors declare that they have no conflicts of interest with the content of this article.
