# Supplementary material for: A split luciferase system for studying coronavirus Mpro dimerization in vitro and in living cells
Source: J Biol Chem. 2025 Nov 4;301(12):110890. doi: 10.1016/j.jbc.2025.110890 (PMC12719657; doi:10.1016/j.jbc.2025.110890)
Supplement: Supplementary material [file mmc1.pdf]

Supporting Information for:

A split luciferase system for studying coronavirus M^{pro} dimerization *in vitro* and in living cells

Renee Delgado[1,*], Jyoti Vishwakarma[1,*], Javier O. Sanlley Hernandez[2], Megan Tansiongco[1,3], Ashley Cuell[1], Agnieszka Dabrowska[1], Rahul Basu[1], Philipp A. M. Schmidpeter[4] ,You Hu[5], Susan E. Tsutakawa[5], Christina B. Cooley[3], Rommie E. Amaro[2] and Reuben S. Harris[1,6,#]

[1] Department of Biochemistry and Structural Biology, University of Texas Health San Antonio, San Antonio, Texas, USA, 78229

[2] Department of Molecular Biology, University of California San Diego, La Jolla, California, USA, 92093

[3] Department of Chemistry, Trinity University, San Antonio, Texas, USA, 78212

[4] Department of Chemistry, The University of Texas at San Antonio, San Antonio, TX, USA, 78249

[5] Molecular Biophysics and Integrated Bioimaging, Lawrence Berkeley National Laboratory, Berkeley, California, USA, 94720

[6] Howard Hughes Medical Institute, University of Texas Health San Antonio, San Antonio, Texas, USA, 78229

* Equal contributions

# Correspondence: rsh@uthscsa.edu

Supplemental materials: Tables S1-S2 and Figures S1-S6

Table S1. Primers used for site-directed mutagenesis.

Primers	Sequences (5'→3') Forward	Sequences (5'→3') Reverse
P132H	ATGTGCTATGCGT**CAT**AATTTTACCATTAAGGGTAGC	TAATGGTAAAATT**ATG**ACGCATAGCACATTGATAAACGCC
S10A	AAAATGGCCTTTCCG**GCC**GGCAAAGTTGAAGGC	TTTGCC**GGC**CGGAAAGGCCATTTTACGAAAGCC
E14A	GGCAAAGTT**GCA**GGCTGCATGGTTCAGGTTAC	CATGCAGCC**TGC**AACTTTGCCGCTCGGAAAGG
C145A	TTTGAACGGTAGC**GCC**GGTAGCGTTGGTTTTAATAT	ACGCTACC**GGC**GCTACCGTTCAAAAAGCTACCCTT
S1A	GCCACCATG**GCC**GGCTTTCGTAAAATGGCCTTTCCGAGC	TTTACGAAAGCC**GGC**CATGGTGGCGGTGGCAAGCTTA
S1D	GCCACCATG**GAC**GGCTTTCGTAAAATGGCCTTTCCGAGC	TTTACGAAAGCC**GTC**CATGGTGGCGGTGGCAAGCTTA
S1Q	GCCACCATG**CAA**GGCTTTCGTAAAATGGCCTTTCCGAGC	TTTACGAAAGCC**TTG**CATGGTGGCGGTGGCAAGCTTA
E166A	TGCTATATGCATCATATG**GCA**TTGCCGACCGGTGTTCAT	CGGCAA**TGC**CATATGATGCATATAGCAGAAAGAGA
E166D	TGCTATATGCATCATATG**GAC**TTGCCGACCGGTGTTCAT	CGGCAA**GTC**CATATGATGCATATAGCAGAAAGAGA
E166Q	TGCTATATGCATCATATG**CAA**TTGCCGACCGGTGTTCAT	CGGCAA**TTG**CATATGATGCATATAGCAGAAAGAGA
E290A	AGCGCTTTGTTGGAAGAT**GCA**TTCACTCCGTTTGAT	AGTGAA**TGC**ATCTTCCAACAAAGCGCTGCCCAAAATGGT
E290R	TGTTGGAAGAT**AGG**TTCACTCCGTTTGATG	CGGAGTGAA**CCT**ATCTTCCAACAAAGCGCTGCC
R4A	CACCGCCACCATGTCTGGCTTT**GAA**AAAATGGCCTTTCC	GGCCATTTT**TTC**AAAGCCAGACATGGTGGCGGTGGCAAGCTTA
R4E	CACCGCCACCATGTCTGGCTTT**GAA**AAAATGGCCTTTCC	GGCCATTTT**TTC**AAAGCCAGACATGGTGGCGGTGGCAAGCTTA

Table S2. **SAXS details and statistics.**

**SAXS Data**	**M^pro + DMSO**	**M^pro + nirmatrelvir**
Simple scattering dataset #	XS5W5HVL	XSMJWVTR
Beamline	ALS SIBYLS 12.3.1	
Data collection mode	MALS-SEC-SAXS	
SEC-SAXS data reduction	BioXTAS RAW	
Low q ($Å^{-1}$)	0.01	
High q ($Å^{-1}$)	0.47	
Low q for analysis ($Å^{-1}$)	0.017	0.012
High q for analysis ($Å^{-1}$)	0.47	0.47
Reciprocal Rg (Å)	26.17	26.29
Reciprocal I(0 (detector units)	138	145
Real space Rg (Å)	26.17	26.15
Real space I(0 (detector units)	138	145
Dmax (Å)	81	80
SAXS MW (kDa)	54.6	58.1
MALS MW (kDa)	58.6	62.8
Theoretical MW of dimer (kDa)	69.5	
Energy (keV)	12	
Temperature (°C)	20	

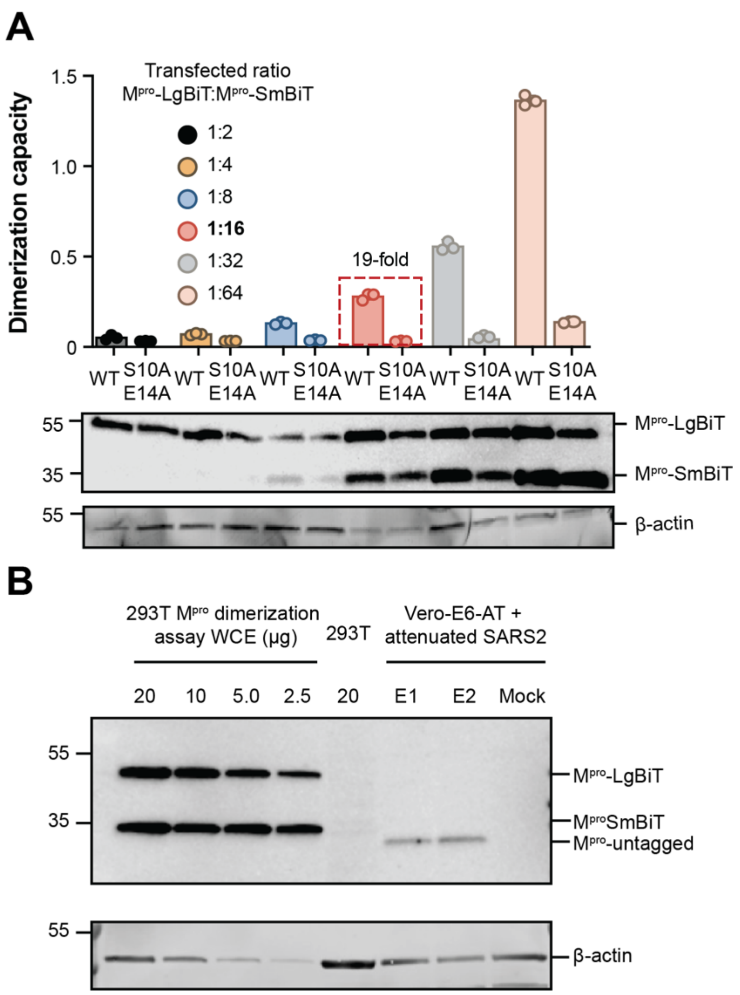

Figure S1. Assay optimization.

(***A***) Bar graphs showing the dimerization capacity (relative luminescence) of M^{pro}-WT and M^{pro}-S10A-E14A constructs in 293T cells. Data are presented as mean ± S.D. from at least 2 independent biological replicates, each done in technical triplicate. Representative anti-M^{pro} immunoblots from a single experiment are shown below each graph with β-actin as a loading control.

(***B***) Immunoblot showing M^{pro} expression levels in whole-cell extracts (WCE) prepared from M^{pro}-LgBiT/M^{pro}-SmBiT(1:16)-transfected 293T cells in comparison to virally expressed protein in Vero-E6-AT cells infected with an attenuated strain of SARS2 (rSARS-CoV-2 Δ3a/7b; PMID: 37191507). Several factors explain the difference in M^{pro} expression levels between these two systems (7-fold between bands in 10 μg versus E1 lanes) including transient transfection versus virus infection, high transfection efficiency (>50%) versus low MOI (<0.01), and virus-mediated cell death.

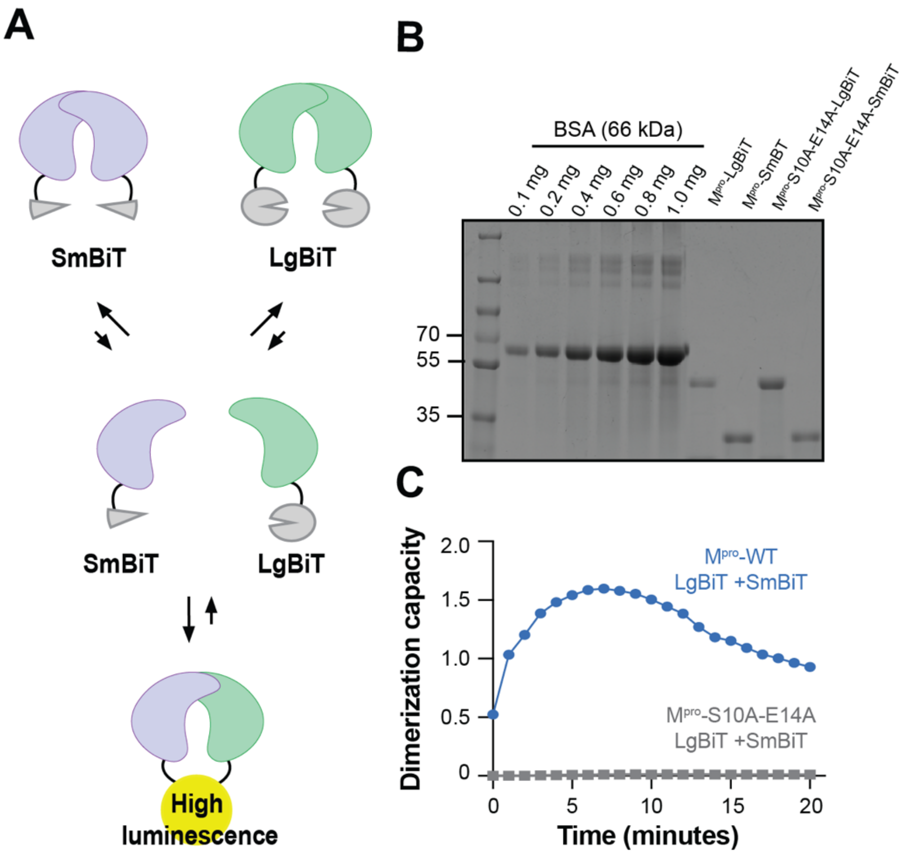

Figure S2. Split-luciferase complementation assay with purified M^{pro} proteins.
(***A***) Schematic of the dimerization assay *in vitro*.
(***B***) Image of a Coomassie-stained SDS-PAGE gel showing the indicated recombinant proteins.
(***C***) Kinetics of dimerization of the indicated proteins over time (luminescent signal x10^5). The drop in luminescence after the peak is likely due to instability of the luciferase substrate fumarizine.

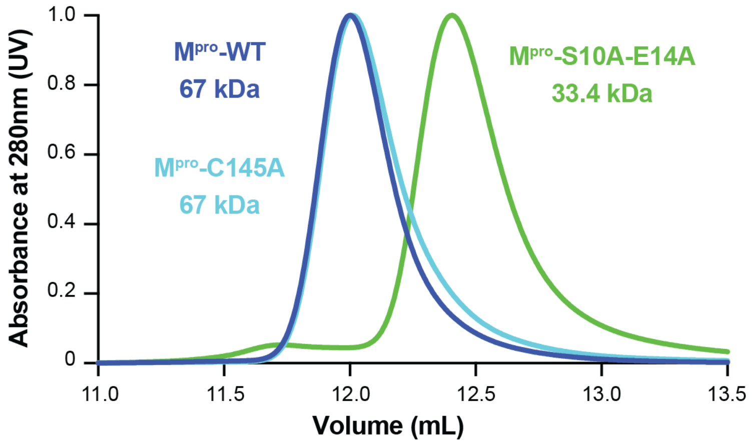

Figure S3. SEC-MALS of WT and mutant M^{pro}.

Elution profiles of the indicated recombinant proteins and their approximate molecular weights.

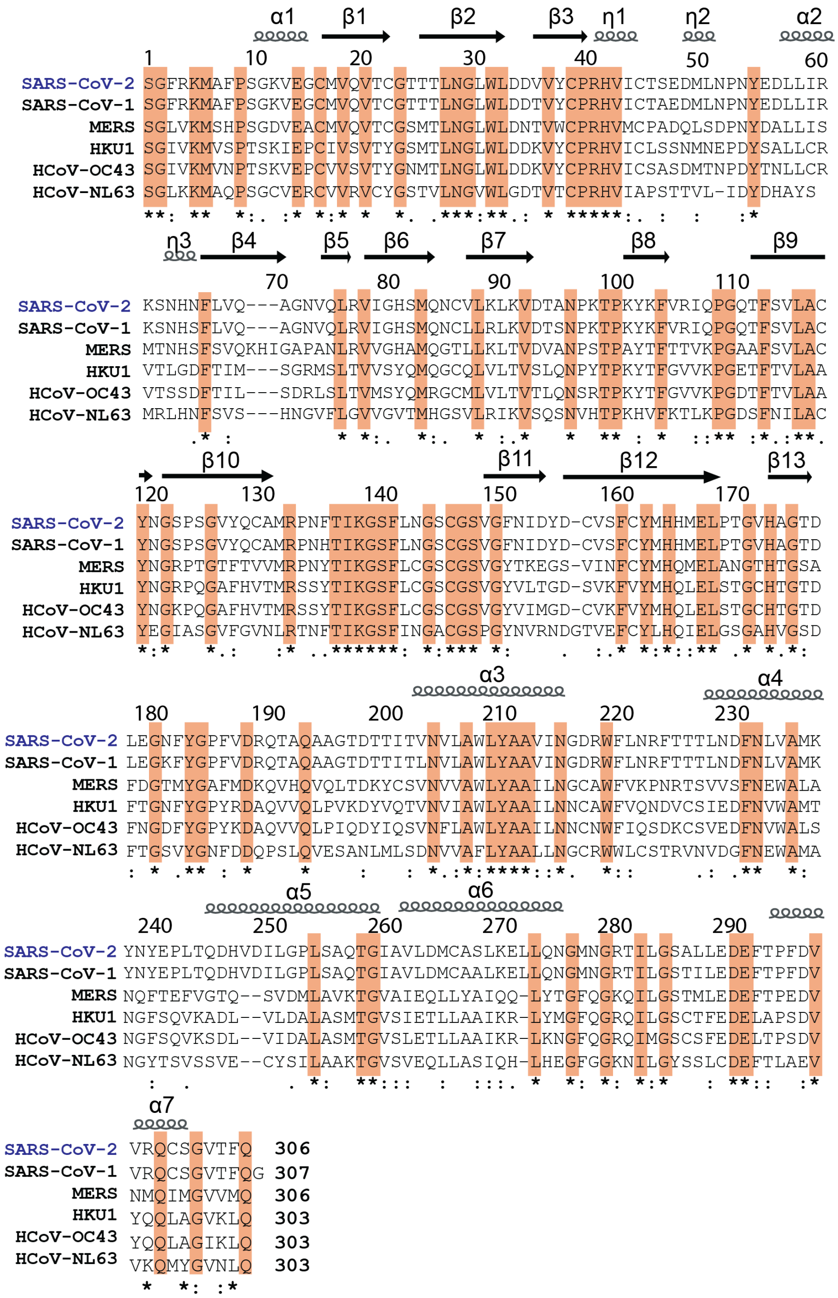

Figure S4. Amino acid alignment of a representative β- and α-coronaviruses.

An alignment of the indicted β- and α-coronavirus main protease amino acid sequences. Conserved residues are highlighted in orange and indicated by asterisks, and similar residues by colons. Secondary structures (α-helices, β sheets, and η-helices) were obtained from PDB structures: 8BFQ (SARS2), 1Q2W (SARS1), 5C3N (MERS), 3D23 (HCoV-HKU1), 9C7W (HCoV-OC43), and 7E6R (HCoV-NL63).

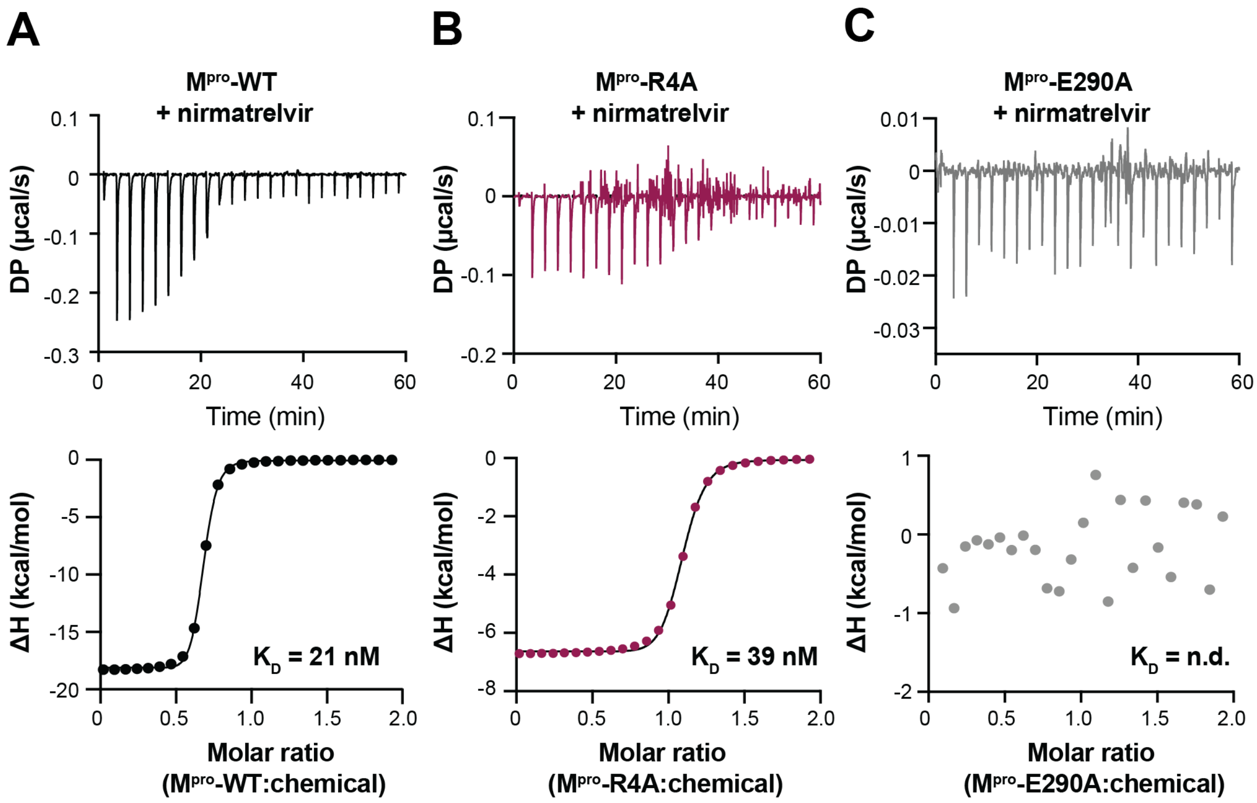

Figure S5. ITC analysis of nirmatrelvir binding to M^{pro} mutants.

(***A***) Untagged WT M^{pro} binding curve with nirmatrelvir.

(***B***) Untagged M^{pro}-R4A binding curve with nirmatrelvir.

(***C***) Untagged M^{pro}-E290A binding curve with nirmatrelvir (n.d., not determinable).

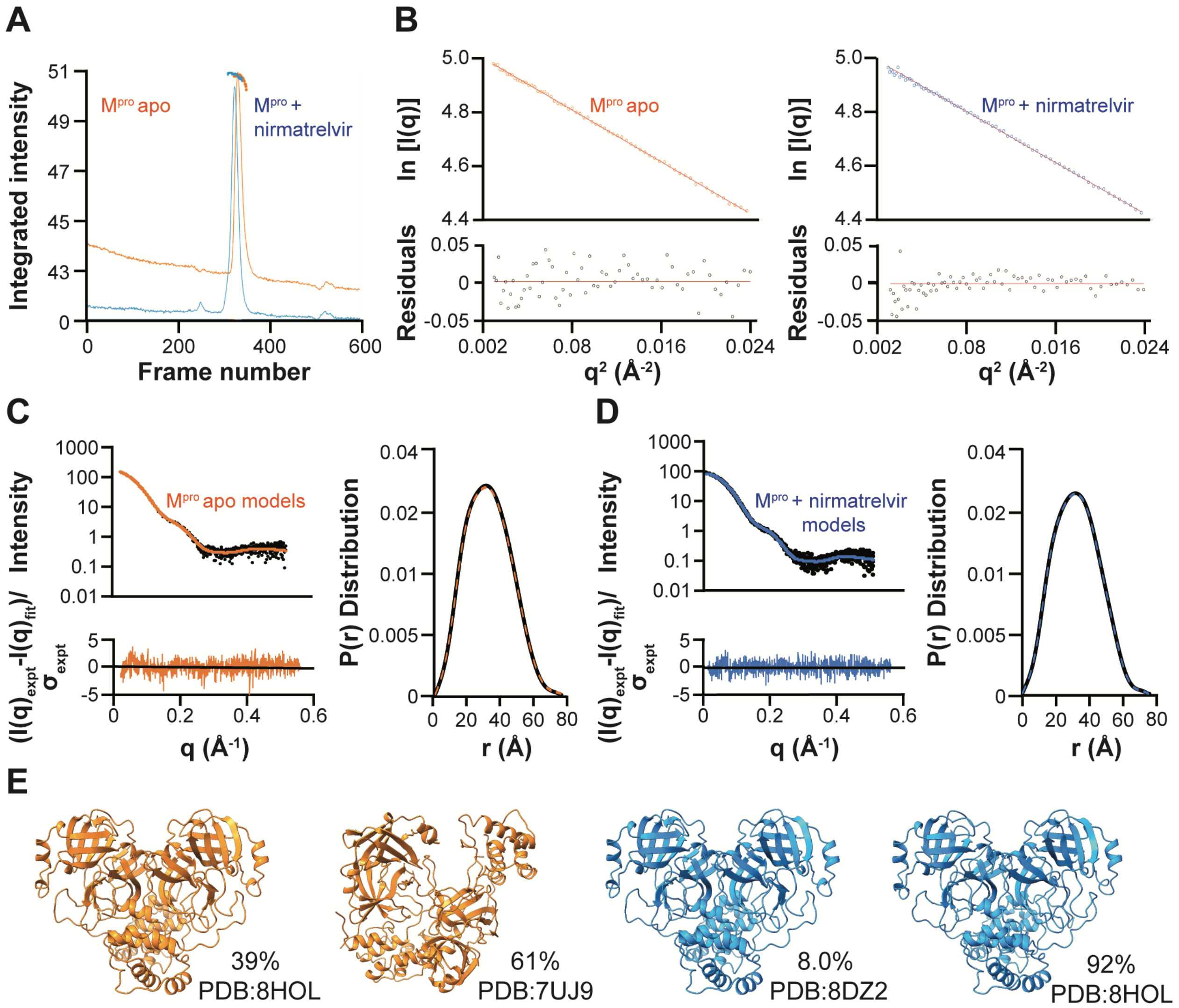

Figure S6. SAXS and structural modeling show nirmatrelvir stabilize M^{pro} dimer in solution. (***A***) SEC profiles from SEC-SAXS of M^{pro} and M^{pro} with nirmatrelvir are consistent with a dimer. A shoulder observed in the M^{pro} apo might indicate the presence of a monomeric species. (***B***) Guinier analysis of the SAXS data is linear (M^{pro} in orange, M^{pro} with nirmatrelvir in blue), consistent with no aggregation. The corresponding residuals are shown in the lower panel. (***C-D***) Reciprocal and real space experimental SAXS intensity profiles compared to theoretical scattering from best-SAXS-fit structural models (M^{pro} apo: orange and M^{pro} + nirmatrelvir: blue).
(***E***) Ribbon schematics of best SAXS-fit models are structurally distinct for M^{pro} dimer, with a loose dimer under apo conditions (orange) and a stabilized dimer bound to nirmatrelvir (blue).
